# Women's Education and Health Inequalities in Under-Five Mortality in Selected Sub-Saharan African Countries, 1990–2015

**DOI:** 10.1371/journal.pone.0159186

**Published:** 2016-07-21

**Authors:** Aristide Romaric Bado, A. Sathiya Susuman

**Affiliations:** 1 Biomedical and Public Health Department, Research Institute of Health Sciences, Ouagadougou, Burkina Faso; 2 Department of Statistics and Population Studies, Faculty of Natural Sciences, University of the Western Cape, Cape Town, South Africa; Institute for Health & the Environment, UNITED STATES

## Abstract

**Background:**

The aim of the study was to analyse trends in the relationship between mother’s educational level and mortality of children under the year of five in Sub-Saharan Africa, from 1990 to 2015.

**Data and Methods:**

Data used in this study came from different waves of Demographic and Health Surveys (DHS) of Sub-Saharan countries. Logistic regression and Buis’s decomposition method were used to explore the effect of mother’s educational level on the mortality of children under five years.

**Results:**

Although the results of our study in the selected countries show that under-five mortality rates of children born to mothers without formal education are higher than the mortality rates of children of educated mothers, it appears that differences in mortality were reduced over the past two decades. In selected countries for our study, we noticed a significant decline in mortality among children of non-educated mothers compared to the decrease in mortality rates among children of educated mothers during the period of 1990–2010. The results show that the decline in mortality of children under five years was much higher among the children born to mothers who have never received formal education—112 points drop in Malawi, over 80 in Zambia and Zimbabwe, 65 points in Burkina Faso, 56 in Congo, 43 in Namibia, 27 in Guinea, Cameroon, and 22 to 15 in Niger. However, we noted a variation in results among the countries selected for the study—in Burkina Faso (OR = 0.7), in Cameroon (OR = 0.8), in Guinea (OR = 0.8) and Niger (OR = 0.8). It is normally observed that children of mothers with 0–6 years of education are about 20% more likely to survive until their fifth year compared to children of mothers who have not been to school. Conversely, the results did not reveal significant differences between the under-five deaths of children born to non-educated mothers and children of low-level educated mothers in Congo, Malawi and Namibia.

**Conclusion:**

The decline in under-five mortality rates, during last two decades, can be partly due to the government policies on women’s education. It is evident that women’s educational level has resulted in increased maternal awareness about infant health and hygiene, thereby bringing about a decline in the under-five mortality rates. This reduction is due to improved supply of health care programmes and health policies in reducing economic inequalities and increasing access to health care.

## Introduction

The mortality rate of children under the age of five years is a key indicator of a child’s well-being, including health and nutrition status. It is also a key indicator of the extent of survival social and economic development interventions that must be implemented for the child’s overall well-being [1]. Millennium Development Goal 4 (MDG 4) calls for reducing the under-five mortality rate by two-thirds between 1990 and 2015. During the last two decades, the Sub-Saharan African countries have witnessed a consistent decline in the under-five mortality [2,3]. The number of deaths among children under-five years of age has decreased annually, from 13 million in 1990 to 9 million in 2007. The global annual rate of reduction has been increasing notably, from 1.2% in 1990–1995 to 2.3% in 1995–2000 and 3.7% in 2000–2005 [1] to 3.9 percent in period 2005–2012.

Tremendous efforts have been made to improve the access to healthcare for children in developing countries, thereby leading to the decline in mortality rates. Efforts have been underway since the early 1980s to strengthen healthcare systems in the developing countries [4,5]. The increase in the supply of primary healthcare facilities and services to the local community through the contracting of community health workers [5–7] has improved the healthcare scenario for children in developing countries. Likewise, certain interventions have greatly contributed towards improving the health of children, thereby reducing infant mortality in countries with limited income. These interventions include malaria prevention through a large scale distribution of impregnated mosquito nets with long-lasting insecticide [8]; indoor residual spraying; and programmes against diarrheal diseases, respiratory infections and malnutrition.

Several countries in Sub-Saharan Africa have implemented user fees reduction or user fees exemption to reduce the overall burden of direct payments for health services, and/or to benefits priority user groups that avail priority services[9]. Pregnant women and children under-five years of age are often the beneficiaries of such fee reduction programmes, which are mainly implemented in countries like Senegal, Ghana, Mali, Niger, Benin, Burkina Faso, Burundi, Kenya, Madagascar, North Sudan, Nepal and Sierra Leone [9]. These user fee policies have certainly contributed towards saving the lives of children in many Sub-Saharan African countries. Studies in Niger [10] and in Burkina Faso [11] have shown that the exemption of fees has resulted in improved usage of healthcare services; reduced economic inequality and under-five mortality rates [12]; and improved access of poor families to basic amenities like health, nutrition, and educational services. Similarly, cash-transfer programmes in several Southern and East African countries also improved survival of children under five years, increased usage of preventive services, improved immunisation coverage, and encouraged healthy behaviours, thereby producing a good outcome [13–17].

Despite these declines and progress, Sub-Saharan African countries are far from reaching the MDG 4. The Sub-Saharan African countries still record the highest under-five mortality rates [18] and over 4.4 million of these deaths are primarily caused due to infectious diseases, which can be avoided by practicing healthy habits [18]. Accounting for only 15% of the world’s population, the Sub-Saharan African countries have more than 41% of children younger than five years, and countries like Nigeria, DR Congo and Ethiopia are among the top 10 countries in the world that record maximum under-five deaths. Although under-five mortality rate and malnutrition are continuing to decline in most Sun-Saharan African countries large inequalities between poor and better-off children exist both between and within these countries[19]. Evidence also shows alarming disparities in under-five mortality rates within countries. Additionally, the risks of under-five mortality increase for children born to uneducated mothers in remote areas or poor households. Survey data show that the under-five mortality rates for the poorest fifth of the population average around twice as high as the average rates for the richest fifth. These inequalities, which appear to be widening, call into question the strategies for child mortality reduction relied upon until date [19].

The variables that are used to measure health inequalities in developing countries include educational [20,21] level of the mother, socioeconomic status [22,23] and place of residence (urban vs. rural) [24]. While recent studies have focused on inequalities related to socioeconomic status, very few recent studies have examined inequalities in under-five deaths and maternal education level. Most of these studies were conducted in the 1980s and early 1990s, during which several Sub-Saharan African countries witnessed the introduction of educational reforms that aimed at increasing the educational level of women and girls. The studies of Caldwell [25,26] and Hobcraft [27] revealed the significance of maternal education in determining of child health, spurring extensive studies in developing countries on the subject. These latter studies also confirmed maternal education to be a very strong and consistent predictor of reduced child morbidity and mortality. The existing literature highlights that female education has been a key to reducing infant and child mortality and fertility rate; increasing consumption of health services; and improving socioeconomic status [28]. Additionally, maternal education increases a mother’s awareness of good health care practices, children’s illnesses, and availability of health services, and hence is considered as a factor that may positively affect a child’s health. The authors [27,28] have argued that education could change attitudes and behaviours of women, thereby enabling them to attain greater autonomy and efficiency. The authors also emphasise on the fact that educated mothers have a greater ability to identify healthcare services for treating their child’s illnesses; higher receptivity to new health-related information; familiarity with modern medical culture; access to financial resources and health insurance; better decision-making power; and increased self-worth and self-confidence.

Several empirical studies [25,26,29–31] have confirmed the positive effects of mother's education on a child’s survival. However, the results of some studies are mixed. A few studies show a very low impact of education on child’s health, while other studies have shown that there is no link between the educational level of mother and a child’s health [32]. This finding implies that although mother's educational level influences the health and survival of the child, the effect may vary from one country to another and from one context to another. In investigating the pathways of influence, research confirms that the causal linkages between maternal education and child’s health are far from clear, and the relationship between these two factors is simply not a reflection of a co-occurrence of education with other socioeconomic variables [27,32,33].

Using data from selected Sub-Saharan countries of several birth cohorts, this study aimed to analyse trends in the relationship between the mother’s educational level and inequalities in under-five mortality over the past two decades in Sub-Saharan African countries (1990 to 2015). This study also aimed to evaluate the effect of maternal educational level on the mortality rate of children under five years of age. The study hypothesises that inequalities in under-five mortality related to mother’s educational level has declined over the last two decades in Sub-Saharan Africa, and the gap in the risks of under-five deaths between children born to *best* educated mothers and children born to *less* educated mothers is narrowing other time.

## Methods

### Data

Huge progress has been made over the last decades to improve the literacy status of adult women around the Sub-Saharan Africa[34]. While gender disparities in adult literacy rates remain wide in Sub-Saharan Africa due to many factors including culture and lack of infrastructures, evidences indicate that some progress has been made at regional and country levels to improve the literacy level of women[34].

**Fig 1:**
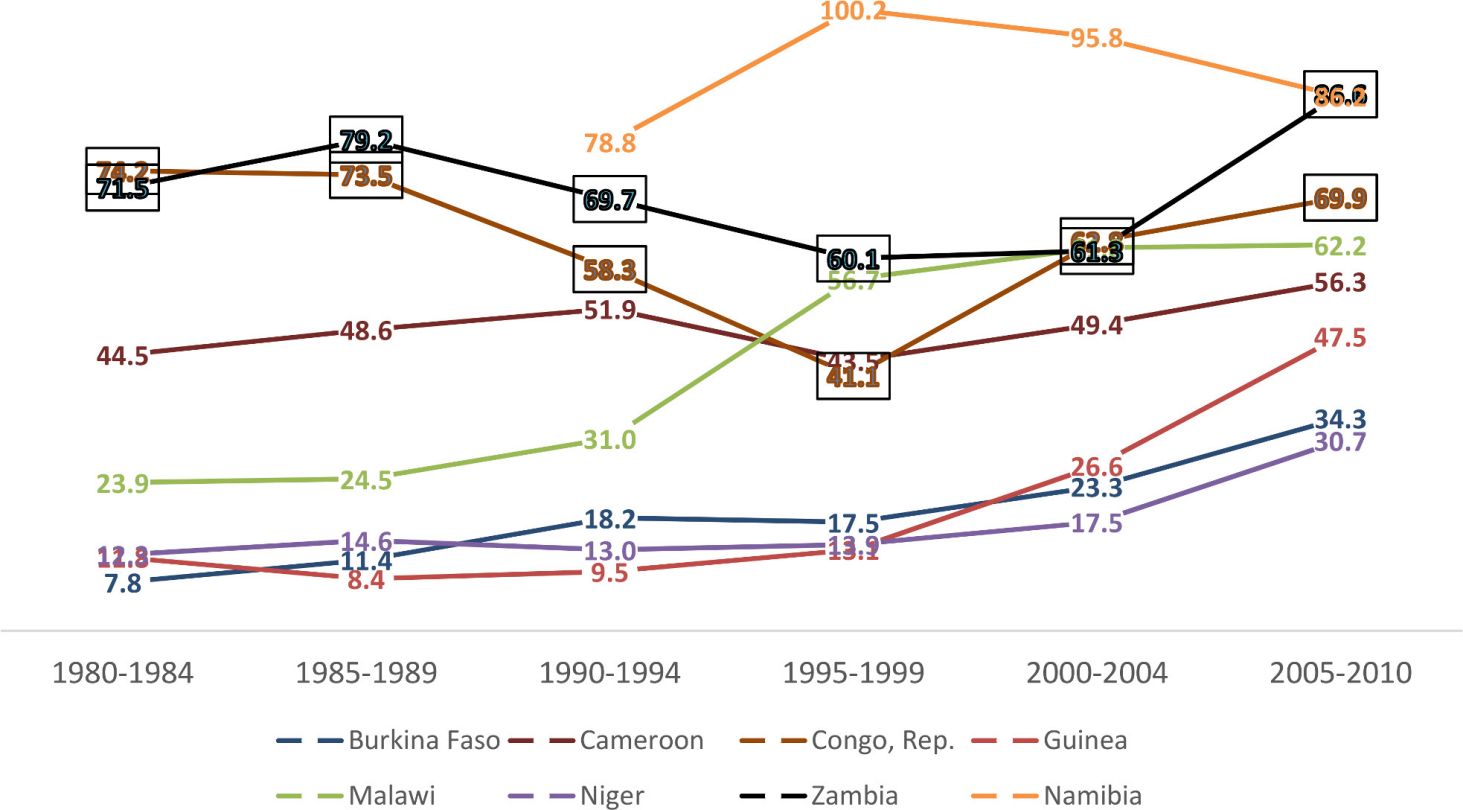
Trend of female primary completion rate (%).

Fig 1 presents the trend of female primary education completion rates in the countries selected for this study. The results show that although the level of women's education has greatly improved during the period 1980–2010, there is a slower increase in the West African countries. The results show that the rate of increase in female education has been 30.7% in Niger, 34.3% in Burkina Faso and 47.5% in Guinea, for the period 2005–2010, compared to other countries that record an increase of above 56%.

Data used in this study come from different waves of DHS of Sub-Saharan countries (**Table 1**), including Burkina Faso, Niger, Guinea, Cameroon, Congo, Malawi, Zambia, and Namibia. The countries were selected according to a sub-regional basis selecting at least two countries from each sub-region of the Sub-Saharan Africa (Western Africa, Central Africa, Eastern and Southern Africa). The selected countries have already realised at least two DHS rounds in the period 1990–2015.

**Table 1:** Selected Sub-Saharan African countries and their DHS study periods.

Sub-Saharan	Country	1990–1999	2000–2009	2010–2015
Western Africa	Burkina Faso	1993	2003	2010
	Niger	1992	2006	2011
	Guinea	1992	2003	2007
Central Africa	Cameroon	1991	2004	2011
	Congo	-	2005	2011
Eastern Africa	Malawi	1992	2000	2010
	Zambia	1996	2007	-
Southern Africa	Namibia	1992	2007	2013

First, DHS waves of survey data of each selected country were pooled, and then data from all the countries were pooled for the purpose of analysis. DHS data were collected using a standardised questionnaire, which was used in all the countries and for different waves of DHS. This offers an advantage for data analysis and comparison of results across countries. This study used birth history data from DHS.

**Table 2**given below presents the number of children under five years and the survival status of each birth cohort by country. The birth history (birth cohort) dataset contained information on the date of birth of all the children born to a woman during her lifetime, starting from the first child to the total number of children born at the time of the survey. Additionally, information on child’s survival (dead or alive) was available in these datasets [35]. Birth histories were collected from a sample of women aged 15–49, at the time of the survey. The complete birth histories (including date of the birth and survival status of each child born to these women until the time of survey) of women aged 15 to 49 years old is considered to be useful data for computing child mortality indicators. DHS data are cross-sectional data, and therefore the survey data represents the entire population. In this context, the information collected during the study included demographic, socioeconomic, and maternal and child health data. The sample design of these surveys is a nationally representative sample and a stratified two-stage cluster design.

**Table 2:** Numbers of children under five years old and the survival status per birth-cohorts per country.

*Country*	**Generation**	**Alive**	**Dead**	**Total**	_5_q_0_
*Burkina Faso*	1986–1989	1909	367	2276	161.2
	1990–1999	10355	1664	12019	138.4
	2000–2009	18497	2062	20559	100.3
	2010–2013	2162	90	2252	40
*Niger*	1986–1989	-	-	-	-
	1990–1999	4166	548	4714	116.2
	2000–2009	13832	1607	15439	104.1
	2010–2013	5748	327	6075	53.8
*Guinea*	1986–1989	-	-	-	-
	1990–1999	4960	790	5750	137.4
	2000–2009	8314	1078	9392	114.8
	2010–2013	3601	252	3853	65.4
*Cameron*	1986–1989	2084	230	2314	99.4
	1990–1999	3652	350	4002	87.5
	2000–2009	13987	1579	15566	101.4
	2010–2013	3251	160	3411	46.9
*Congo*	1986–1989	-	-	-	-
	1990–1999	-	-	-	-
	2000–2009	9527	725	10252	70.7
	2010–2013	3647	142	3789	37.5
*Malawi*	1986–1989	1470	380	1850	205.4
	1990–1999	10900	1816	12716	142.8
	2000–2009	27505	2670	30175	88.5
	2010–2013	2015	90	2105	42.8
*Zambia*	1986–1989	2832	563	3395	165.8
	1990–1999	12250	2096	14346	146.1
	2000–2009	8717	870	9587	90.7
	2010–2013	-	-	-	-
*Namibia*	1986–1989	1439	136	1575	86.3
	1990–1999	5215	314	5529	56.8
	2000–2009	6687	406	7093	57.2
* *	2010–2013	3614	156	3770	41.4

### Ethics statement

The datasets used in this study were obtained from the DHS program thanks to the authorisation received to download the dataset on the website (https://dhsprogram.com/data/available-datasets.cfm).

### Variables

The dependent variable of this study is the child survival status (alive or dead) during the duration of the survey. The wealth indexes, the mother’s educational level, and the place of residence (rural/urban) are the main explanatory variables that assess the inequalities in child mortality. Households were grouped into five categories of wealth index (poorest, poor, middle, rich and richest). In terms of maternal education, we categorised the mothers into three groups (not attended school, 1–6 years of education and more than 6 years of education).

The other covariate variables included sex and birth order of the child, parity, mother’s age during childbirth and the size of the household.

### Statistical methods

#### Descriptive and multivariate analyses

First, the study used concentration index as well as absolute and relative ratios of mortality rates to measure inequality in health. The concentration index ranges between -1 and 1, and a negative value in this study means the deaths are more concentrated among children born to *less* educated mothers. Two logistic regression models were built to estimate the effect of mother educational level on the probability of a child to die before reaching his fifth birthday: Model 1 gave the unadjusted effect and Model 2 gave the adjusted effect of the co-variables.

#### Decomposition analysis

This study used the method proposed by Buis [36] which decomposes the total association between a categorical, discrete or continuous exposure variable, and an outcome from a direct effect and an indirect effect. The decomposition method proposed by Buis is described in his article published in 2010. In the current study, it is assumed that the dependent variable Y represents the child’s mortality and X represents the mother’s educational level. X is the main dependent variable by which we seek to quantify the direct effect, and Z is the indirect effect of the effect of all other covariates in our study. OR is considered as the risk of child to die before reaching his fifth birthday.

$$\ln(OR_{x=1,z|x=1})-\ln(OR_{x=0,z|x=0})=\ln(OR_{x=0,z|x=1})-\ln(OR_{x=0,z|x=0})+\ln(OR_{x=1,z|x=1})-\ln(OR_{x=0,z|x=1)}$$ (1)

From Eq 1, the total effect (ln(*OR*_*x* = 1,*z*|*x* = 1_)−ln(*OR*_*x* = 0,*z*|*x* = 0_)) is the sum of the direct effect (ln(*OR*_*x* = 0,*z*|*x* = 1_)−ln(*OR*_*x* = 0,*z*|*x* = 0_)) and direct effect (ln(*OR*_*x* = 1,*z*|*x* = 1_)−ln(*OR*_*x* = 0,*z*|*x* = 1)_))

After transformation of the Eq (1), we obtain the Eq (2) below where:

$\ln(\frac{OR_{x=1,z|x=1}}{OR_{x=0,z|x=0}})$ is the total effect, $\ln(\frac{OR_{x=0,z|x=1}}{OR_{x=0,z|x=0}})$ and $\ln(\frac{OR_{x=1,z|x=1}}{OR_{x=0,z|x=1}})$ are respectively the indirect and direct effect.

$$\ln(\frac{OR_{x=1,z|x=1}}{OR_{x=0,z|x=0}})=\ln(\frac{OR_{x=0,z|x=1}}{OR_{x=0,z|x=0}})+\ln(\frac{OR_{x=1,z|x=1}}{OR_{x=0,z|x=1}})$$ (2)

## Results

### Trend of mother’s educational level and under-five mortality inequalities

**Fig 2:**
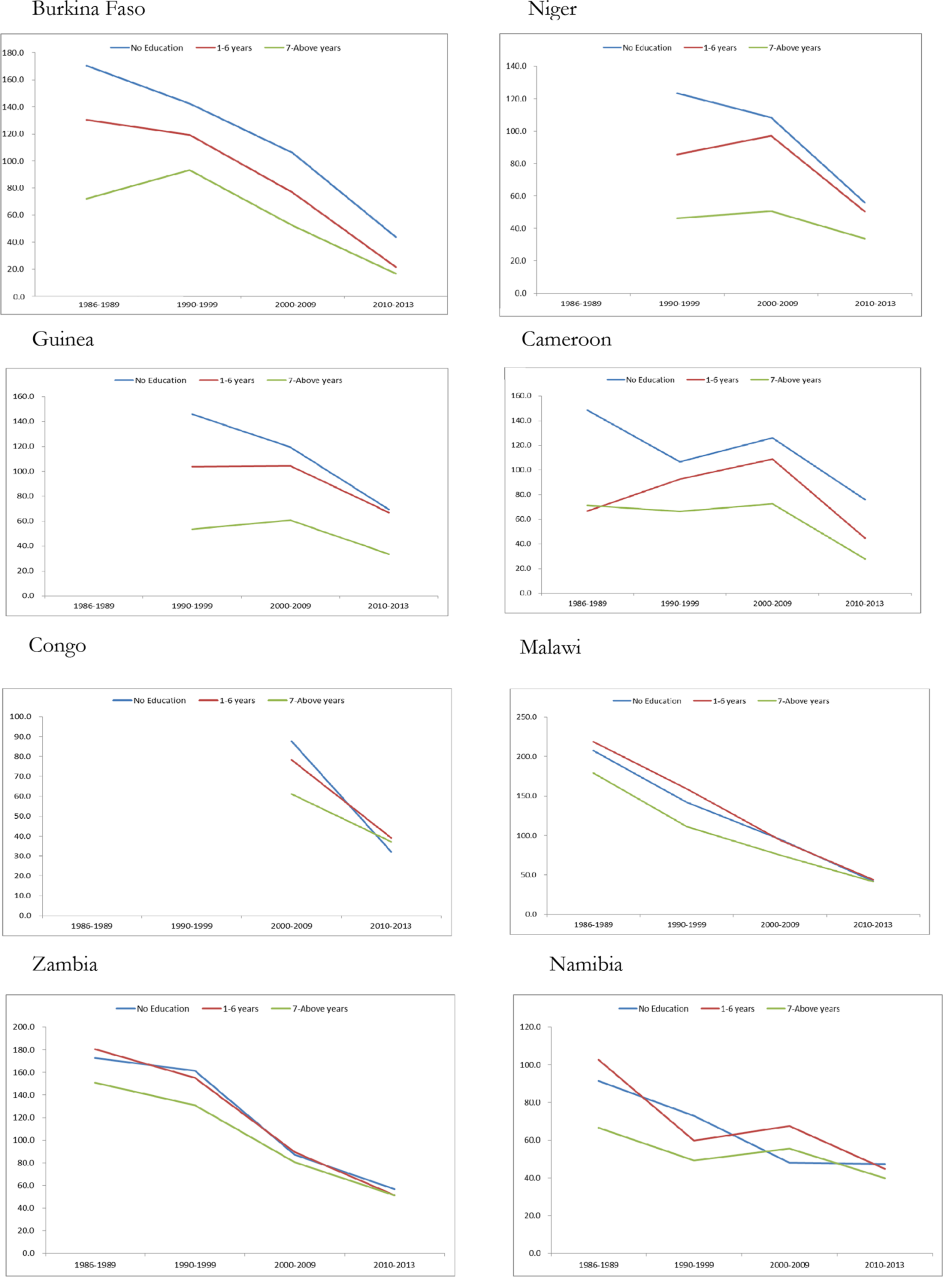
Trends of under-five mortality rate by birth cohort and the education level of the mother.

Fig 2 depicts the trend of under-five mortality rate and maternal education level by birth cohorts of children. The results reveal that, in general, children of mothers who did not attend school have a higher rate of death compared to mothers with formal education. However, mortality rate differentials are reduced from the older birth-cohort of children (1986–1989) to the younger birth-cohort of children in each country. The results also show a variation among the countries (Fig 2). Cameroon, Burkina Faso, Niger and Guinea are countries showing large inequalities in mortality by education level of the mother. Indeed, in these countries, the concentration index is less than -0.10 (-0.19 for the birth cohort of 1986–1989, -0.10 for the birth cohort of 1990–1999 and -0.11 to -0.20 for birth cohort of 2000–2009 and birth cohort of 2010–2013). This shows that the high concentration of deaths is among children born to mothers without aby formal education.

The absolute differences in under-five mortality rates between children of more educated mothers (seven years and above) and children of uneducated mothers in Cameroon are 99.4, 87.5, 101.5 and 47 points respectively for birth cohorts of 1986–1989, 1990–1999, 2000–2009 and 2010–2013. In Burkina Faso, the absolute differences have decreased from 98.4 points for children of the birth-cohort of 1986–1989 to 27 points for children of the youngest birth cohort (born after 2009).

These differences account for less than 25 points in Namibia. The under-five year mortality concentration indices for children born between 1990 and 1999 in Niger and Guinea are a proximally -0.05, whereas it is -0.04 for those in the 2000–2010 birth cohort. These results show a high concentration of risk of mortality among children of non-educated mothers. The trend of the under-five mortality rate and the level of education of mothers in Malawi have moved from a situation of high mortality rate with a high concentration of mortality risk among children of non-educated mothers to a situation of low mortality rate. The inequalities of under-five mortality by mother’s educational level have greatly decreased in Zambia from the oldest birth cohort to the youngest birth-cohort.

### Mother’s educational level and reduction of under-five mortality

**Table 3**presents the comparative results of the under-five mortality rates of birth cohort according to different levels of mother’s education. The results show that the decline in mortality of children under five years was much higher among the children of mothers who had not been to school. The findings show a 112 points drop in Malawi, over 80 in Zambia and Zimbabwe, 65 points in Burkina Faso, 56 in Congo, 43 in Namibia, 27 in Guinea and Cameroon, and 22 to 15 in Niger. With regards to mothers with 1–6 years of education, the declines in mortality rates for children under five years were less compared to the groups of children born to non-educated mothers. The findings reported significant differences in under-five mortality comparison between the older birth cohort and younger birth cohort. These differences were 125 points for Malawi, 91points for Zambia, 54 points for Burkina Faso, and 40 points for Congo. However, the study reported an upward trend for Cameroon (-42 points), Niger (-11 points), and Guinea (-8) points. A downward trend was noticed for children born to educated mothers. There was a significant difference in decline in under-five mortality rates between old birth cohort and the young birth cohort 104.4 points difference in Malawi, more than 40 points difference in Zambia and 24 points in Congo. The declines were not significant in Burkina Faso, Niger, Guinea, Cameroon and Namibia.

**Table 3:** Comparison of the mortality rates of children under five years old by birth-cohorts in the selected countries.

Country	Mother’s educational level	1986–1989	1990–1999	2000–2009	2010–2013	Difference (per 1000)	*z-test*	*P-value*
Burkina Faso (1986–1989 vs 2000–2009)	No Education	170.5	142.4	105.9	43.7	64.6	8.49	0.001
	1–6 years	130.4	119.4	76.8	21.6	53.6	2.93	0.003
	≥7 years	72.1	93.4	52.4	16.7	19.7	0.87	0.386
Niger (1990–1999 vs 2000–2009)	No Education		123.3	108.5	55.8	14.8	2.6	0.009
	1–6 years		85.7	97.1	50.3	-11.4	-9.88	0.001
	≥7 years		46.4	50.6	33.8	-4.2	-0.24	0.809
Guinea (1990–1999 vs 2000–2009)	No Education		146.1	119.6	69.3	26.5	4.36	0.001
	1–6 years		103.7	104.5	67.1	-0.8	3.11	0.002
	≥7 years		53.6	60.7	33.4	-7.1	-0.43	0.668
Cameroon (1986–1989 vs 2000–2009)	No Education	148.6	106.5	126.2	75.9	22.4	1.79	0.073
	1–6 years	66.6	92.7	108.9	44.6	-42.3	-3.69	0.001
	≥7 years	71.4	66.3	72.5	27.9	-1.1	0.48	0.629
Congo (2000–2009 vs 2010–2013)	No Education			87.84	32	55.84	3.55	0.001
	1–6 years			78.46	39.17	39.29	-28.64	0.001
	≥7 years			61.23	37.08	24.15	-32.43	0.001
Malawi (1986–1989 vs 2009)	No Education	207.4	142.3	95.1	42.3	112.3	9.75	0.001
	1–6 years	218.8	159.5	94.2	43.9	124.6	8.99	0.001
	≥7 years	179.5	111.6	75.1	41.5	104.4	7.48	0.001
Zambia (1986–1989 vs 2000–2009)	No Education	172.8	161.2	87.1	56.6	85.7	6.001	0.001
	1–6 years	180.6	155.3	89.7	51.3	90.9	9.38	0.001
	≥7 years	150.7	130.9	80.7	51.1	70	8.2	0.001
Namibia (1986–1989 vs 2000–2009)	No Education	91.5	73	48.1	47.3	43.4	2.75	0.006
	1–6 years	102.6	59.8	67.5	44.8	35.1	2.81	0.005
	≥7 years	66.6	49.3	55.5	40	11.1	1.12	0.263

### Effects of mother’s educational level on inequalities in under-five mortality

**Tables 4 & 5**shows the multivariate results. The study aimed to examine the gross effect of maternal education on child mortality, and subsequently obtain the net effect after controlling for other covariates. An analysis of gross effects in Model 1 (Table 4) shows that the mother’s educational level is significantly associated with the likelihood of under-five mortality in the selected countries of our study. Model 2 presents the adjusted OR.

**Table 4:** Effects of selected factors assisted with under five mortality (Model 1: gross effects).

**Factors**	**Burkina Faso**	**Cameroun**	**Malawi**	**Zambia**	**Niger**	**Namibia**	**Guinea**	**Congo**
	Model 1	Model 1	Model 1	Model 1	Model 1	Model 1	Model 1	Model 1
	OR(95%CI)	OR(95%CI)	OR(95%CI)	OR(95%CI)	OR(95%CI)	OR(95%CI)	OR(95%CI)	OR(95%CI)
**Mother’s educational level**								
**No Education**	1	1	1	1	1	1	1	1
**1–6 years**	0.7***(0.6–0.8)	0.8***(0.7–0.8)	0.9(0.9–1.0)	0.9* (0.8–1.0)	0.8*(0.7–1.0)	1.1(0.9–1.3)	0.8**(0.7–0.9)	0.9(0.7–1.2)
**≥7 years**	0.5***(0.4–0.6)	0.5***(0.5–0.6)	0.7***(0.6–0.7)	0.8***(0.7–0.8)	0.4***(0.3–0.6)	0.8**(0.7–0.9)	0.4***(0.3–0.5)	0.7*(0.6–0.9)
**Constant**	0.1***(0.1–0.1)	0.1***(0.1–0.1)	0.1***(0.1–0.1)	0.1***(0.1–0.1)	0.1***(0.1–0.1)	0.1***(0.1–0.1)	0.1***(0.1–0.1)	0.1***(0.1–0.1)
**N**	37,096	25,276	46,842	41,082	26,203	17,953	18,985	14,036

*** p<0.001

** p<0.01

* p<0.05

OR: Odds Ratios

CI: confidence interval

N = Number of observations

**Table 5:** Effects of selected factors assisted with under five mortality (Model 2: Adjusted effects).

**Factors**	**Burkina Faso**	**Cameroon**	**Malawi**	**Zambia**	**Niger**	**Namibia**	**Guinea**	**Congo**
	Model 2	Model 2	Model 2	Model 2	Model 2	Model 2	Model 2	Model 2
	OR(95%CI)	OR(95%CI)	OR(95%CI)	OR(95%CI)	OR(95%CI)	OR(95%CI)	OR(95%CI)	OR(95%CI)
**Mother’s educational level**								
**No Education**	1	1	1	1	1	1	1	1
**1–6 years**	0.9*(0.8–1.0)	0.8**(0.8–0.9)	1.1(1.0–1.1)	1(0.9–1.1)	1(0.9–1.1)	1.1(0.9–1.3)	0.9(0.8–1.1)	1(0.8–1.2)
**≥7 years**	0.8*(0.6–1.0)	0.7***(0.6–0.8)	0.8***(0.7–0.9)	0.8**(0.8–1.0)	0.7*(0.5–0.9)	1(0.8–1.3)	0.6***(0.4–0.7)	0.8(0.6–1.0)
**Birth-cohort**								
**1986–1989**	1	1	1	1		1		
**1990–1999**	0.8*(0.7–1.0)	0.9(0.8–1.1)	0.7***(0.6–0.7)	0.5***(0.5–0.6)	1	0.7***(0.6–0.9)	1	
**2000–2009**	0.6***(0.5–0.7)	1(0.9–1.2)	0.4***(0.3–0.4)	0.3***(0.3–0.3)	0.9*(0.8–1.0)	0.7**(0.6–0.9)	0.8***(0.7–0.9)	1
**2010–2013**	0.2***(0.2–0.3)	0.5***(0.4–0.6)	0.2***(0.1–0.2)	-	0.5***(0.4–0.5)	0.5***(0.4–0.6)	0.5***(0.4–0.6)	0.5***(0.4–0.6)
**Parity**								
**03-Jan**	1	1	1	1	1	1	1	1
**06-Apr**	1.4***(1.2–1.5)	1.2**(1.1–1.4)	1.2***(1.1–1.3)	1.2***(1.1–1.4)	1.4***(1.2–1.6)	1.4***(1.2–1.6)	1.4***(1.2–1.6)	1.2*(1.0–1.5)
**≥7**	1.7***(1.5–1.9)	1.6***(1.4–1.9)	1.4***(1.2–1.6)	1.3**(1.1–1.4)	1.8***(1.6–2.1)	1.5**(1.2–2.0)	2.0***(1.7–2.3)	1.4*(1.1–1.9)
**Mather’ age at birth**								
**< = 19**	1	1	1	1	1	1	1	1
**20–34**	0.7***(0.6–0.8)	0.7***(0.7–0.8)	0.8***(0.7–0.9)	0.8**(0.8–0.9)	0.7***(0.6–0.8)	0.8*(0.6–1.0)	0.8**(0.7–0.9)	0.8(0.6–1.0)
**35–49**	0.7***(0.6–0.8)	0.7***(0.5–0.8)	0.8***(0.7–0.9)	0.7***(0.6–0.9)	0.7***(0.5–0.8)	0.8(0.6–1.0)	0.7**(0.6–0.9)	0.9(0.7–1.3)
**Birth Interval**								
**1st Birth**	1	1	1	1	1	1	1	1
**<24 Months**	1.3***(1.1–1.5)	1.4***(1.2–1.6)	1.1(1.0–1.2)	1.1(1.0–1.3)	1(0.8–1.1)	1.4*(1.1–1.7)	1(0.8–1.2)	1.1(0.8–1.5)
**24–35 Months**	0.8***(0.7–0.9)	0.8***(0.7–0.9)	0.6***(0.5–0.7)	0.7***(0.6–0.7)	0.7***(0.6–0.8)	0.9(0.8–1.2)	0.7***(0.6–0.9)	0.8(0.7–1.1)
**36–47 Months**	0.5***(0.5–0.6)	0.6***(0.5–0.8)	0.5***(0.4–0.6)	0.6***(0.5–0.7)	0.5***(0.4–0.6)	0.8(0.6–1.1)	0.6***(0.5–0.7)	0.7*(0.6–1.0)
**≥48 Months**	0.4***(0.3–0.4)	0.6***(0.5–0.8)	0.5***(0.5–0.6)	0.6***(0.5–0.7)	0.4***(0.3–0.5)	0.9(0.7–1.1)	0.4***(0.3–0.4)	0.7**(0.5–0.9)
**Multi-Birth**								
**Twin**	1	1	1	1	1	1	1	1
**Single**	0.3***(0.2–0.3)	0.3***(0.3–0.4)	0.2***(0.2–0.3)	0.3***(0.2–0.3)	0.2***(0.2–0.3)	0.2***(0.2–0.3)	0.3***(0.3–0.4)	0.2***(0.2–0.3)
**Child' sex**								
**Female**	1	1	1	1	1	1	1	1
**Male**	1.1*(1.0–1.2)	1.1**(1.0–1.2)	1.2***(1.1–1.2)	1.2***(1.1–1.3)	1.1*(1.0–1.2)	1.1(1.0–1.3)	1.2***(1.1–1.3)	1.1(0.9–1.3)
**Residence place**								
**Urban**	1	1	1	1	1	1	1	1
**Rural**	1.2* (1.0–1.3)	1(0.9–1.1)	1.1(1.0–1.2)	0.9**(0.8–1.0)	1.4***(1.2–1.7)	0.9(0.8–1.1)	1.1(0.9–1.3)	1(0.8–1.2)
**Socioeconomic status**								
**Poorest**	1	1	1	1	1	1	1	1
**Poorer**	1(0.9–1.1)	0.8***(0.7–0.9)	1.1(1.0–1.2)	1(0.9–1.1)	1.2**(1.1–1.4)	1.1(0.9–1.4)	0.9(0.8–1.1)	1.1(0.9–1.4)
**Middle**	1(0.9–1.1)	0.8**(0.7–0.9)	1.1(1.0–1.2)	1(0.9–1.2)	1.2*(1.0–1.4)	1(0.8–1.2)	0.9(0.8–1.0)	1.2(1.0–1.6)
**Richer**	0.9(0.8–1.0)	0.8**(0.6–0.9)	1.1(1.0–1.2)	1(0.9–1.2)	1.3***(1.1–1.5)	1(0.8–1.2)	0.8**(0.7–0.9)	1.1(0.8–1.5)
**Richest**	0.8***(0.7–0.9)	0.6***(0.5–0.8)	0.9(0.8–1.0)	0.7***(0.6–0.9)	1(0.8–1.2)	0.7*(0.5–0.9)	0.7***(0.5–0.8)	0.9(0.6–1.3)
**Constant**	0.9(0.7–1.2)	0.6***(0.4–0.7)	1.2(1.0–1.5)	1(0.8–1.3)	0.5***(0.4–0.7)	0.4***(0.3–0.6)	0.6***(0.5–0.8)	0.4***(0.2–0.6)
**N**	37,096	25,276	46,842	34,674	26,203	17,953	18,985	14,036

*** p<0.001

** p<0.01

* p<0.05

OR: Odds Ratios

CI: confidence interval

N = Number of observations

In all these cases, children of more educated mothers are not likely to die before the age of five (OR = 0.4 in Guinea and in Niger, OR = 0.5 in Burkina Faso and Cameroon, OR = 0.7 in Malawi and Congo, and OR = 0.8 in Zambia and Namibia) compared to children born to mothers without any formal education.

With regards to Burkina Faso (OR = 0.7), Cameroon (OR = 0.8), Guinea (OR = 0.8) and Niger (OR = 0.8), children of mothers with 0–6 years of education are about 20% more likely to survive until their fifth birthday compared to children of non-educated mothers. There are no significant differences between children of mothers with 0–6 years of education and those of mothers who have not been educated who die before their 5th birthday in Congo, in Malawi and in Namibia.

The results (Model 2 in Table 5) depict the net effects (the adjusted OR after controlling the confounding variables. As in the case of Model 1, the results vary by country. With the exception of Namibia and Congo, where the mother’s educational level loses its significance, the mother’s educational level is significantly associated with under-five mortality in other countries. While there is a 50% to 20% likelihood that children born to educated mothers might not die before their fifth birthday, it might not be the case for under-five children of mothers without any formal education.

**Table 5**shows that although the effect of maternal education declined in Burkina Faso while controlling the effect of the covariate, the impact of maternal education on under-five mortality still remains significant. However, there is no significant difference in mortality between children of mothers with 1–6 years of education and children born to non-educated mothers. Variables such as the birth cohort, maternal age during childbirth, the birth interval, multi-births, the child's sex, and place of residence are proved to be significantly associated with the mortality of children under-five years. Children born between 1990 and 1999 (OR = 0.8; 95%CI (0.7; 1.0), 2000–2009 (OR = 0.6, 95%CI = 0.5, 0.7) and after 2009 (OR = 0.2 95% CI (0.2; 0.3)) have a lower risk of dying before five years than children born before 1990. Children born to mothers aged 20–34 were (OR = 0.7; 95%CI (0.6; 0.8)) 30% less likely to die before their fifth birthday compared to children born to mother aged less than 20 years. The children delivered at a single birth (OR = 0.3 95%CI (0.2; 0.3)) were 70% less likely to die before their fifth birthday compared to children born from multiple births. Male children (OR = 1.1, 95%CI = (1.0; 1.2)) and those rural residents (OR = 1.2, 95%CI (1.0; 1.3)) are more likely to die before their fifth birthday than female children and those living in urban areas.

With regards to Niger, the results of the final model show that maternal education has dropped slightly but still remains significant after the introduction of control variables. It appears that children born between 2000 and 2009 (OR = 0.9, 95%CI (0.8, 1.0)), and post 2009 (OR = 0.5, 95%CI (0.4; 0.5)) were less likely to die before the age five compared to children born between 1990 and 1999. Also, the risk of mortality reduced for children born to f mothers who delivered between 20–34 of age (OR = 0.7, 95%CI (0.6, 0.8)) and maintained more than 24 months of birth interval. However, this was not the case for children born to young mothers (≤19 years) and children of first-time mothers. The results also show that, compared to twins, non-twins (OR = 0.2, 95%CI (0.2; 0.3) had about 80% less risk of dying before reaching their fifth birthday.

Like mother’s educational level, the variables that were significantly associated with under-five mortality rate in Guinea included birth cohort, parity, and maternal age at delivery, inter-birth interval, and type of birth (twin vs. single), the child's sex and socioeconomic status. The observed relationships are similar compared to those found in Burkina Faso and Niger. In Cameroon, the variables significantly associated with mortality were birth cohort, parity of the mother, the mother's age at delivery, inter-birth interval, type of birth, the sex of the child and, the socioeconomic status. While in Congo, birth cohort, the type of delivery, and socioeconomic status were significantly associated with the mortality of children under five years. In Malawi and Zambia, in addition to the mother’s educational level, the birth cohort, inter-birth interval, the type of delivery, and the child's sex were significantly associated with under-five mortality. The maternal age at birth, birth interval, multi or single birth, socioeconomic statuses were determinants of mortality in children under five years in Namibia.

### Direct and indirect effects of mother’s educational level on under five mortality

**Table 6 and Fig 3**show the decomposition results of the direct and indirect effect of mother’s education on under-five mortality. The results show that the direct effect of mother's educational level varies between 35% (in Namibia) to 79% (in Congo). The direct effect of mother's educational level is not significant in Namibia and Congo. In the case of Namibia, the education of the mother is not a determinant of mortality in children under five years of age.

**Fig 3:**
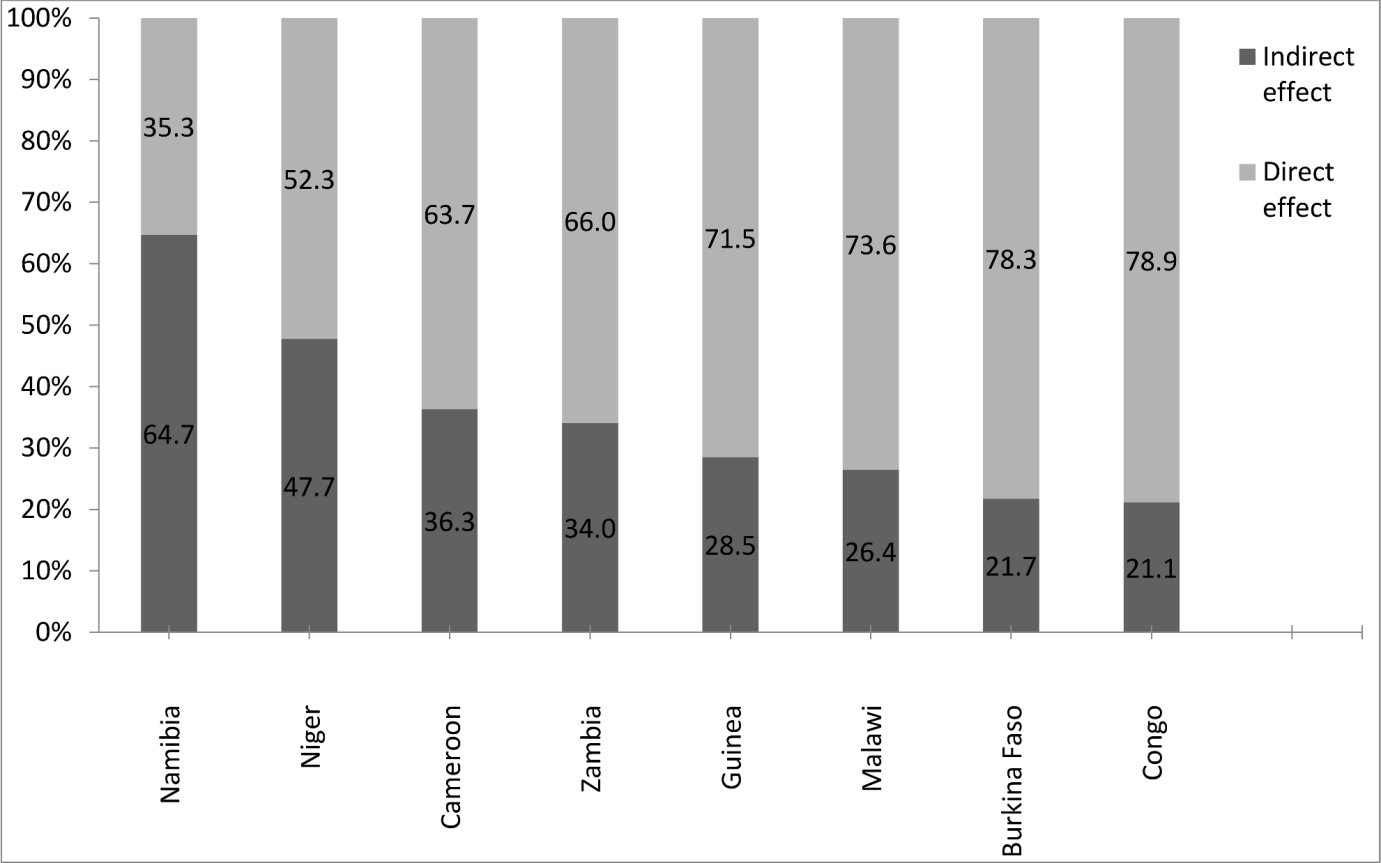
Direct and indirect effect of the education of the mother and under-five mortality.

**Table 6:** Decomposition of Direct and Indirect effect of mother’s educational level on under five years old mortality.

	Coef.	Std. Err.	% of Effect	P>z	95% CI
Burkina Faso					
≥7 years /No education					
Total	-0.69	0.09	100.0	0.001	-0.87; -0.50
Indirect Effect	-0.15	0.04	21.7	0.001	-0.22; -0.08
Direct **effect** of mother educational level	-0.54	0.10	78.3	0.001	-0.73; -0.34
Niger					
≥7 years /No education					
Total	-0.83	0.11	100.0	0.001	-1.05; -0.62
Indirect Effect	-0.40	0.04	47.7	0.001	-0.48; -0.32
Direct **effect** of mother educational level	-0.44	0.12	52.3	0.001	-0.67; -0.20
Guinea					
≥7 years /No education					
Total	-0.93	0.16	100	0.001	-1.24; -0.62
Indirect Effect	-0.26	0.04	28.5	0.001	-0.35; -0.12
Direct **effect** of mother educational level	-0.66	0.17	71.5	0.001	-0.99; -0.33
Cameroon					
≥7 years /No education					
Total	-0.66	0.06	100.0	0.001	-0.78; -0.55
Indirect Effect	-0.24	0.04	36.3	0.001	-0.32; -0.17
Direct **effect** of mother educational level	-0.42	0.07	63.7	0.001	-0.56; -0.29
Congo					
≥7 years /No education					
Total	-0.29	0.12	100	0.013	-0.52; -0.06
Indirect Effect	-0.06	0.04	21.1	0.153	-0.15; 0.02
Direct **effect** of mother educational level	-0.23	0.13	78.9	0.065	-0.48; 0.02
Malawi					
≥7 years /No education					
Total	-0.38	0.04	100.0	0.001	-0.45; -0.30
Indirect Effect	-0.10	0.01	26.4	0.001	-0.13; -0.07
Direct **effect** of Mother educational level	-0.28	0.04	73.6	0.001	-0.36; -0.20
Zambia					
≥7 years /No education					
Total	-0.24	0.06	100	0.001	-0.36; -0.12
Indirect Effect	-0.08	0.03	34.0	0.002	-0.13; -0.03
Direct **effect** of mother educational level	-0.16	0.07	66.0	0.018	-0.29; -0.03
Namibia					
≥7 years /No education					
Total	-0.24	0.09	100.0	0.006	-0.41; -0.07
Indirect Effect	-0.16	0.03	64.7	0.001	-0.22; -0.09
Direct **effect** of mother educational level	-0.09	0.10	35.3	0.408	-0.29; 0.12

However, more than three-fourths difference in mortality was observed among children of educated mothers, with seven years of education, and children of non-educated mothers. The differences accounted were 78.3% in Burkina Faso, 73.6% in Malawi and 71.5% in Guinea. This difference is greater than half for Zambia (66.0%), Cameroon (63.7%) and Niger (52.3%). These differences could be due to the direct effect of the educational level of the mother.

## Discussion

The study results clearly show the influence of maternal education on under-five mortality while decomposing the direct and indirect effect of maternal education on mortality of children under five years. The results of our study have shown that the trend of mortality rates by level of mother's education varies from one country to another and we did not get the same trends in all the countries. The trend of under-five mortality by level of mother’s education is similar in Burkina Faso, Niger and Guinea high mortality among children of non-educated mothers, low mortality risk among children of educated mothers (≥7 years schooling), and an intermediate position for children of mothers with 1-6years of education. The trend for other countries is not consistent with this finding. The heterogeneity of the results reveals the importance of unobserved factors which may affect maternal education and child health simultaneously [37]. Additionally, the inconsistency in findings also highlights the importance of contextual effects for explaining the demographic events such as under-five mortality [38,39]. These include the health policies implemented across selected countries; the presence, access and affordability of healthcare services; the availability of community services; a mother’s health status and awareness.

Country specific results confirm the existence of a difference in mortality rates between children of mothers who have been to school and children of mothers without formal education. However, these results varied across countries. The results confirm an inverse relationship between mother’s educational level and the risks of under-five mortality [26,27,40]. With the exception of Namibia, the results showed that children of educated mothers (seven years or more of education) are less likely to die before reaching their fifth birthday compared to children of less educated mothers. These results are in line with the results of previous studies on infant mortality in developing countries [29, 30, 40,41].

Our study also analysed the trend of the under-five mortality of birth cohorts of children born between 1986 and 2013. The results showed a significant decline in mortality among children of mothers without formal education (between 14 points in Niger and Malawi 112 points), while the decrease was less pronounced among children born to mothers with 1–6 years of education (-42 points in Cameroon to 124 points in Malawi), and among children of mothers with seven or more years of schooling. The largest decline among children of non-educated mothers could be explained by the reduction of geographical and financial barriers to accessible healthcare for children under-five year of age in many countries.

The remoteness of health centres is identified as an unfavourable factor for accessing to healthcare services for children, especially in rural areas [42–45]. The current study also identifies the need to reduce delivery costs of healthcare services for promoting the use of maternal and child services, especially for children from poor households [46, 47]. The multivariate results, after controlling for other variables, showed that the effect of educational level of the mother is an important determinant of child mortality in several countries. These analyses also showed the importance of variables such as the generation of child birth, parity of the mother, the mother's age at delivery, inter-birth interval, the type of birth, the sex of the child and the household socioeconomic status.

Our study has some limitations in addition to the limitations often assigned to DHS data. These limitations include event omissions; misreporting of ages and dates of birth and death, especially, for new-borns; and selectivity bias owing to the lack of DHS data on survival or death of children born to mothers who had died at the time of the survey. Additionally, mother's educational level variable represents only the educational level of mothers at the time of the interview, which cannot be reflective of mother’s education at the time of childbirth. It is therefore possible that the educational level of a mother during childbirth might be slightly different than her educational level at the time of the survey. However, we believe that the bias is relatively small and will not impact the results. In this study, we did not pretend to take into account all the determinants of child mortality. Therefore, the study does not consider education level of parents, ethnicity, religion and administrative region, assistance at delivery, and complete or uncertain vaccination.

## Conclusions

The study demonstrates that mothers’ educational attainment is an important determinant of child mortality, and the results confirmed that children of more educated mothers have lesser risk of dying before their fifth birthday compared to children born to mothers without formal education. The analysis of the trend in mortality educational level of the mother showed a significant reduction in mortality gaps between children of mothers with more than seven years of education and children of non-educated mothers. These observed reductions are certainly the result of programmes and policies designed for reducing unequal access to health care and improving the supply of health care, especially, to the most underprivileged communities. If universal access of women to school could induce a reduction in under-five mortality rates in Sub-Saharan countries, it is clear that it should be accompanied by access to policies and healthcare programmes, which can reduce the gaps between children of educated mothers and non-educated mothers.

## Supporting Information

S1 File Demographic and Health Surveys data agreement. (DOCX)
